# Clinico-pathological correlation: Teaching aspects, avenues, and advances

**DOI:** 10.30476/JAMP.2021.89846.1387

**Published:** 2022-01

**Authors:** SUNIL JAIN

**Affiliations:** 1 Department of Paediatrics, Command Hospital (Northern Command), India

**Keywords:** Sciences, Microbiome, Genomics, Systems biology

## Abstract

**Introduction:**

Explosion in scientific knowledge of our understanding of diseases, diagnostics, and targeted therapeutics all possible & advantageously with Clinico-pathological correlation (CPC). A narrative of evolution from the past to the present & the novelty in the future is exciting, with pragmatic presentation of important recent developments & interesting research directions, for ‘evolving excellence’.

**Methods:**

Analysis of the guidance was produced by leading medical education institutions and an in-depth search on Google Scholar, Medline, and PubMed for CPC. We thoughtfully formulated ways of incorporation of CPC for teaching and training of medical students at different levels. We put available policies, evidence, and personal experience gleaned in proper perspective.

**Results:**

The four HPs (i) Historical perspective… Tracing the progress, the important developments extending the physician’s range are clinical correlation with the outgivings of chemical analysis and biological experimentation. (ii) Holistic professionalism: Integrating Basic and Clinical Sciences. Knowledge for practice and knowledge applications in practice are two pillars of medical education. Learning opportunities provisioning should enable linking of theory and practice. (iii) The happening perfect: Clinic-Pathological Conference. Problem-based learning is highly effective. A clinical case presentation with deliberation on all aspects is useful for all. The denouement should include research developments and desired research directions. (iv) Heightened professionalism: Progress features promise exciting future. Chemistry of life and disease has been evolving manifold, opening new vistas. Microbiome research results for clinically actionable microbe-host relationships identification are in foresight. Genomic and proteomic approach to human disease is leading to new understanding of pathogenesis for clinical strategies. Systems Biology studies living organisms with their cellular or molecular network components holistically to predict response to perturbations. ‘Systems pathobiology’ is energising avenues for early diagnosis and advancing clinical applications for halting progress for better medical practice.

**Conclusions:**

Teaching CPC for all round understanding will lead to all knowledgeable proficient medical practitioners.

## Introduction

*“To study the phenomena of disease without books is to sail an uncharted sea, while to study books without patients is not to go to sea at all”.*

Sir William Osler, 

Professor of Medicine, Oxford, UK

Knowledge and experience are the essence of medical education and lead to professional expertise and excellence. Progress in medical sciences has been phenomenal, and our understanding of the phenomenon of disease, the aetio-pathogenesis, the presentations, the progress, the care and cure have all progressed from molecular level to societal interventions. Amidst all this, clinico-pathological correlation assumes an increasingly important role. All these advances have advantageous applications in surgical sciences as well. The diagnostic specialties of pathology and laboratory services are fundamental to a fully functional surgical service. A narrative of progress to the present state and novel advancement avenues in future should guide progress towards greater professionalism. 

The enduring values of the medical profession are stated as: *“No greater opportunity, responsibility, or obligation can fall to the lot of a human being than to become a physician. In the care of the suffering, [the physician] needs technical skill, scientific knowledge, and human understanding…”* ( 1 ). There has been an explosion in scientific knowledge of our understanding of diseases, diagnostics, and targeted therapeutics. All this should be accompanied by evolution of expertise in skills for its application. Hence, the need for putting things in perspective for teaching and training of future medical practitioners.

## Methods

Analysis of the guidance was produced by the leading medical educational institutions and evidence from an in-depth search on Google Scholar, Medline and PubMed for CPC. We thoughtfully formulated ways of incorporation of CPC for teaching and training of medical students at different levels. We put available policies, evidence, and personal experience gleaned in proper perspective.

## Results

The pathways, implementation, potential impacts, and progressive improvements possible with CPC are presented within the context of the ‘4HPs’:

### (i) Historical perspective

Giovanni Battista Morgagni (1682-1771) in his lecture "Nova institutionum medicarum idea (New training medicarum idea)" presented the educational project for the "medicus perfectissimus". In this proposal, the medical course curriculum had to include studies on religious and human law, logic and rhetoric, mathematics, philosophy, chemistry, botany, pharmacy and therapy. Importantly, it included an in-depth study of morphology along with its pathological aspects. Morgagni's educational method proposed adherence to empirical data. It encouraged search for the truth through observations and experiments ( 2 ). Morgagni taught thousands of medical students from many countries at the University of Padua for 56 years as a Professor of Anatomy. He is rightly regarded as the father of pathological anatomy and a pioneer of modern medicine.

In the early 19^th^ century, the forerunners of modem medicine were the French schools, with Corvisart, Laennec, and Louis. In particular Louis exerted a strong influence with his statistical approach to medical problems. Later in the century, Germany attracted some of the best of the younger minds. It was here that in universities practical laboratory teaching was first established. In these laboratories the sciences of chemistry, physics, pathology, physiology, and eventually bacteriology reached a mature status through the efforts of Liebig, Helmholtz, Virchow, Muller, Cohnheim, and Ludwig ( 3 ). 

The Flexner report reformed medical education in the United States ( 4 ). It importantly stated: *“Modern medicine is an attempt to fight the battle against disease, most advantageously for the patient. …… A professional education in medicine is more difficult, more trying, more responsible than a college education”*. The proper basis of medical education in the Flexner report stated: *“…… the stethoscope, the microscope, the correlation of observed symptoms with the outgivings of chemical analysis and biological experimentation, enormously extend the physician’s range”*. These still hold true. All this is best summarised as:

*“Macroscopic picture and microscopic details, Complement each other, and Contribute enormously to rationale practice”*.

### (ii) Holistic professionalism: Integrating Basic and Clinical Sciences

Knowledge for practice and knowledge applications in practice are two pillars of medical education. As per the Flexner report, completion of a basic science curriculum was required before initiating the clinical curriculum. In a workshop on “The Roles of Academic Health Centers in the 21st Century,” Dr. Hundert, Dean University of Rochester School of Medicine and Dentistry, commented that: *“this produced an unintended side effect of implying that basic science is put aside when entering into clinical care—the very opposite of what should be conveyed”* ( 5 ). The double helix curriculum weaving the clinical strand and the basic science strand throughout is the most suitable. The interest in why and how pathological processes are happening, the causation mechanisms, and clinical manifestations should provide insights for focussed, early, and energetic fruitful interventions. 

Another advantage is bringing the basic science faculty into the hospital. In this regard Dr. Hundert commented: *“The effect on the faculty is profound. When the virologist talks to the students about human papilloma virus and the potential for a vaccine to prevent cancer, it happens after the students have seen women with cervical cancer. Students become hungry for information on the basic sciences. Additionally, the virologist interacts with the gynecologist and the oncologist to jointly design new translational research programs and clinical interventions, so faculty members interact in different ways”* ( 5 ). Bedside clinics with bedside comprehensive education including basics and advances in real time is robust teaching.

The Medical Council of India (MCI) has been proactive in this regard. The latest undergraduate curriculum states that the Indian Medical Graduate is required to have the following competency at the time of graduation: *“demonstrate knowledge of normal and abnormal human structure, function and development from a molecular, cellular, biologic, clinical, behavioural and social perspective. It emphasizes de-compartmentalisation of disciplines so as to achieve both horizontal and vertical integration in different phases”* ( 6 ). 

Similarly at post-graduate level the general objectives state that the student should have sufficient understanding of the basic sciences relevant to the concerned speciality. Training programmes in basic medical sciences include exposure to the applied aspects of the subject relevant to clinical specialities. Those of clinical disciplines include practical training in diagnosis; medical and surgical treatment; training in the basic medical sciences; as well as in allied clinical specialities ( 7 ). Practical training is always fruitful.

Super-specialization training programme is on the same pattern as for M.D. / M.S. in clinical disciplines and include practical training, e.g., advanced diagnostic, therapeutic and laboratory techniques, relevant to the subject ( 7 ).

The General Medical Council (GMC), UK, ‘Standards for medical education and training for undergraduates for promoting excellence’, clearly state provisioning of: *“learning opportunities that integrate basic and clinical science, enabling them to link theory and practice”*. The GMC standards for postgraduate training programmes for doctors require clinical placements providing the opportunity to work and learn with other members on the team to support inter-professional multidisciplinary working ( 8 ). Integration of causative pathways and clinical results will result is comprehensive energetic care. All this is best summarised as:

*“Ensuring multi-disciplinary skills and systematic synchronization for excellence”*.

### (iii) The happening perfect: Clinic-Pathological Conference

The Clinic-Pathologic Conferences (CPCs) are big educational events and with best learning experiences. These should continue to be frequent. At the beginning of the 20^th^ century, at Harvard Medical School, Walter Cannon and Richard Cabot had inaugurated the CPC ( 9 ). The CPC consists of presentation of the case, diagnostic data, discussion of differential diagnosis, logically narrowing down to few selected probable diagnoses and eventually telescoping to the target - the final diagnosis, followed by its brief discussion. The presentation of present state of practice and its discussion will result in pertinent felt needs enunciation for progress.

Scientific educational evolution has contributed immensely to the academic value of the CPC sessions. This is with newer techniques, greater objectivity, and introduction of standardized methods of examination. The demands of the medical and surgical super-specialities can be targeted and addressed. More refinements are expected in the future ( 10 ). The denouement should include research developments and desired research directions.

Problem-based learning is highly effective as per medical education research. So, the CPCs focused on clinical problems, and should provide a wonderful learning experience ( 11 ). The CPC clinical case presentation with deliberation on all aspects and discussion on diagnosis, including its approach and management attributes, make it an excellent educational experience. All members of the medical community, from medical student to senior clinician, participate. The MCI’s ‘The Postgraduate Medical Education Regulations’ recommends CPCs for training programmes for specialization in clinical disciplines. We feel these are beneficial for all, including the undergraduates. Good things learnt in formative years lead to great-going in future practice. All this is best summarised as: 

*“CPCs impart knowledge and skills, aiming at most comprehensive clinical problem solving with clinical presentation and pathological correlation”*.

### (iv) The progress and future: Heightened professionalism

*“Cellular pathology is not an end if one cannot see any alteration in the cell. Chemistry brings the clarification of living processes nearer than does anatomy. Each anatomical change must have been preceded by a chemical one”* ( 12 ).

Rudolf Virchow

Chemistry of life and disease has evolved manifold, opening new vistas. Evolution from the past to the present has been encouraging and the future exciting, all towards excellence. Understanding of genetics, epigenetics, and stromal influences in disease are a few examples of recent progress.

The field of microbiome research is progressing with great strides with clinically actionable microbe-host relationships identification in foresight. Resulting comprehensive clinical applications should be all advantageous. Presently, research is exploring (i) gut microbiome effects on different aspects of human health; (ii) skin health improvements by modulating the cutaneous microbiota, study of antimicrobial peptides produced by probiotics as well as resident bacteria; (iii) microbial ecology in different tumours and focus on the roles of microorganisms in cancer formation, development and response to treatments.

Genomic and proteomic approach to human disease is leading to new understanding of pathogenesis. The expanding knowledge base of associations between genetic variation and human disease is the essence of Genomics. It will aid in the development of effective strategies for early diagnosis and treatment. Genomics is increasingly used for understanding, classifying and treating human cancers, etc. Inquisitive and innovative interest in Genomics tools and techniques will lead the way to better associate Genomics and the medicine of tomorrow. Proteomics is improving our understanding of how protein structure determines function. Usefulness is towards new diagnostic and prognostic tests, targeted therapeutics, accelerating drug developments with efficacy and minimal toxicity. Databases freely accessible to academic communities are available. An excellent example is ‘Human Protein Reference Database (HPRD)’, an object database that integrates a wealth of information, important in the function of human proteins in health and disease ( 13 ). Clinico-Pathological correlation is interesting with these advances, and will provide avenues for best clinical strategies. 

Of particular interest is Systems Biology. It is the holistic study of living organisms or their cellular or molecular network components to predict their response to perturbations. Its concepts can be applied readily to human disease and therapy. The field of systems pathobiology includes genetic or environmental perturbations producing disease and drug perturbations restoring normal system behaviour. The promise of Systems Biology is predictive and preventative medicine leading to personalized medicine. With therapeutic strategies tailored to individual needs, Systems Biology will fundamentally transform society ( 14 ). All this should be with efficiency and towards excellence.

Systems pathobiology can be importantly used to revise, refine, and rationalize the definition of human diseases and their classification, guided by the growing body of evidence of causation, course, and corrections. The classification of human disease used in all medical textbooks, beginning in the 19^th^ century, derives from the correlation between pathologic analysis and clinical syndromes. Refinements in this are possible with inclusion of sensitivity in defining preclinical disease and incorporation of the molecular and genetic determinants of patho-phenotype into the conventional classification scheme. Systematic analysis for sophisticated advancements is regularly required ( 15 ). Systems pathobiologic approach and analyses will lead to a drastic revision of the way human disease is defined and treated. Prevention is always better than cure. Avenues for early diagnosis and advances in clinical applications for halting progress are likely to be energised with ‘systems pathobiology’ and result in better medical practice.

## Conclusions

Teaching clinic-pathological correlation and its practical training will lead to excellence. Medical teaching for sophisticated competency should include and integrate CPC in all its avenues and advancements, and at all levels. This is for our definitive contributions for conquest of diseases. All this is best summarised as:

*“Deviations from health and disease, The causation, the extent, the effects, the care, & the cure, All round understanding with teaching clinic-pathological correlation, for All knowledgeable proficient medical practitioners”*.

## Acknowledgement

Thankful to the formulators of all regulatory policies and guidelines and to the authors of the references quoted.

## Ethics

The article comprises views on medical education policies and published evidence. It does not involve any intervention or experimentation on any living beings. 

## Conflict of Interest:

None Declared.
